# Entry of PIP3-containing polyplexes into MDCK epithelial cells by local apical-basal polarity reversal

**DOI:** 10.1038/srep21436

**Published:** 2016-02-22

**Authors:** Cuifeng Wang, Edwin de Jong, Klaas A. Sjollema, Inge S. Zuhorn

**Affiliations:** 1 University Medical Center Groningen, University of Groningen, Department of Cell Biology, Antonius Deusinglaan 1, 9713 AV Groningen, The Netherlands

## Abstract

The polarized architecture of epithelium presents a barrier to therapeutic drug/gene carriers, which is mainly due to a limited (apical) internalization of the carrier systems. The bacterium *Pseudomonas aeruginosa* invades epithelial cells by inducing production of apical phosphatidylinositol-3, 4, 5-triphosphate (PIP3), which results in the recruitment of basolateral receptors to the apical membrane. Since basolateral receptors are known receptors for gene delivery vectors, apical PIP3 may improve the internalization of such vectors into epithelial cells. PIP3 and nucleic acids were complexed by the cationic polymer polyethylenimine (PEI), forming PEI/PIP3 polyplexes. PEI/PIP3 polyplexes showed enhanced internalization compared to PEI polyplexes in polarized MDCK cells, while basolateral receptors were found to redistribute and colocalize with PEI/PIP3 polyplexes at the apical membrane. Following their uptake via endocytosis, PEI/PIP3 polyplexes showed efficient endosomal escape. The effectiveness of the PIP3-containing delivery system to generate a physiological effect was demonstrated by an essentially complete knock down of GFP expression in 30% of GFP-expressing MDCK cells following anti-GFP siRNA delivery. Here, we demonstrate that polyplexes can be successfully modified to mimic epithelial entry mechanisms used by *Pseudomonas aeruginosa*. These findings encourage the development of pathogen-inspired drug delivery systems to improve drug/gene delivery into and across tissue barriers.

Gene therapy requires safe and efficient carriers that deliver the nucleic acids (DNA, RNA) into cells. In order to achieve this aim, a number of obstacles needs to be overcome by the gene delivery system. At the cellular level, multiple membranous barriers need to be passed, namely the plasma membrane, the endosomal membrane, and for DNA the nuclear membrane. Moreover, for *in vivo* applications the gene delivery system needs to be stable in biological fluids until it reaches the target cells. Historically, epithelia, that line the cavities and surfaces of organs, were considered easy targets for gene delivery, because of their direct accessibility via topical and enteral administration. However, epithelia turn out to form huge barriers for gene delivery because they display multiple features that discourage the uptake of gene vectors.

Epithelial monolayers consist of polarized cells that are connected through tight junctions, that separate the plasma membrane of the cells into an apical and basolateral domain. The apical surface, that faces the lumen, is strengthened by actin filaments close to the plasma membrane. The tight junctions, together with the junctions that are formed between neighboring cytoskeletal networks through desmosomes, prevent the paracellular transport of all molecules, with the exception of very small polar molecules^1^,^2^. This way, the epithelial cell monolayer forms a physical barrier, thereby preventing the penetration of harmful substances including pathogens. In addition, the innate immune system broadly protects the epithelium against the interaction with pathogens and also stimulates the adaptive immune response^3^. Despite these defense mechanisms, opportunistic pathogens like the bacterium *Pseudomonas aeruginosa* have established ways to invade the polarized epithelium. It was recently shown that when *P.aeruginosa* binds to the apical surface, basolateral proteins become recruited to the apical surface by activation of the PI3K/Akt pathway, leading to the formation of basolateral domains at the apical surface^4^. At the site of bacterium binding, protrusions are formed that are enriched in phosphatidylinositol-3, 4, 5-trisphosphate, basolateral proteins, and actin. The integrity of the overall cell polarity in this process is maintained, which suggests that *P.aeruginosa* induces the movement of basolateral proteins to the apical surface via transcytosis rather than diffusion^5^.

In mammalian cells, phosphoinositides play a key role in determining cell polarity. Phosphatidylinositol-4, 5-bisphosphate (PIP2) primarily localizes to the apical surface, whereas phosphatidylinositol-3, 4, 5-trisphosphate (PIP3) is found at the basolateral membrane^6^. Insertion of exogenous PIP3 at the apical surface results in the rapid transformation of regions of the apical surface into a membrane with the composition of the basolateral surface by basolateral-to-apical transcytosis^7^. Since the basolateral membrane is prone to endocytosis of viral (e.g. Ad, AAV) and non-viral vectors (e.g. LF2k)^8^,^9^,^10^,^11^, the presence of basolateral domains at the apical surface may improve the endocytic capacity of the epithelium for gene delivery vectors that are luminally applied. Here, we hypothesize that local apical-basal polarity reversal in polarized epithelial cells may facilitate the entry of gene delivery vectors without barrier disruption.

Polyethylenimines (PEIs) are promising non-viral polymeric gene carriers, that can condense nucleic acids into nanoscale complexes through electrostatic interaction^12^. In general, negatively charged nucleic acids show poor uptake in cells, whereas positively charged PEI-nucleic acid complexes, i.e., PEI polyplexes, significantly improve nucleic acid internalization via endocytosis. PEIs with a high cationic charge density also serve to facilitate the endosomal escape of the nucleic acids by the so-called “proton sponge effect”^13^, which represents an important step in the gene delivery process that critically determines transfection efficiency^14^. In addition, PEI has been used for PIP3 delivery into cells^15^. Therefore, we investigated whether a ternary complex of PEI, nucleic acids, and PIP3 could be used to enhance gene delivery into polarized epithelial cells. Ternary complexes of PEI, DNA and poly (α-glutamic acid) or heparin have previously been made to reduce the overall positive charge of the complexes in order to avoid the undesired interaction with negatively charged serum proteins, which may lead to recognition and clearance by the reticuloendothelial system^16^,^17^. Here, it is investigated whether PIP3-containing PEI polyplexes induce the recruitment of basolateral receptors to the apical cell surface in MDCK cells. In addition, PEI polyplexes with and without PIP3 are compared for their cellular binding and uptake, intracellular trafficking, endosomal escape, and transfection efficiency.

## Results and Discussion

### Apical incubation of MDCK cell monolayers with PIP3/Histone recruits basolateral receptors to the apical surface

**Figure 1:**
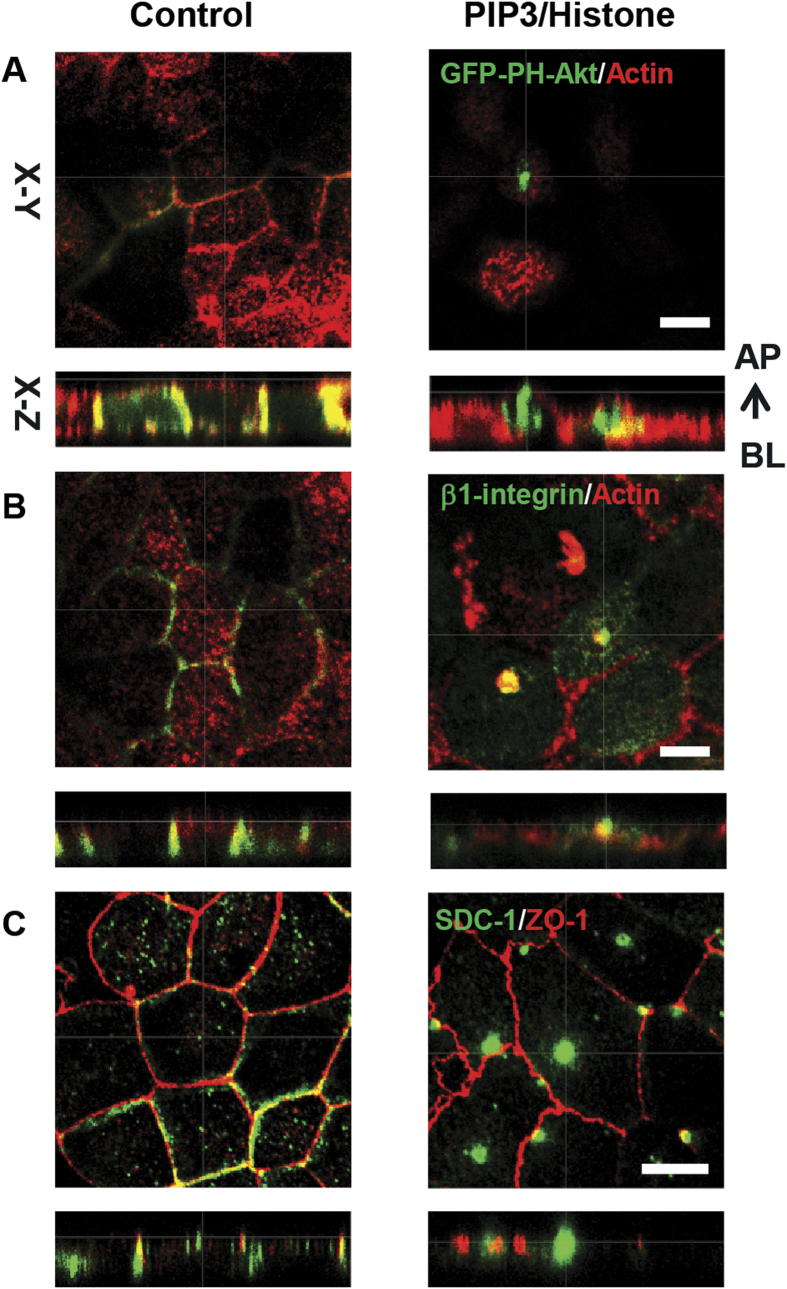
Apical incubation of MDCK cells with PIP3/Histone induces recruitment of basolateral receptors. (**A**) Polarized MDCK cells stably expressing GFP-PH-Akt (green) were treated with PIP3/Histone complex, or without treatment (control), for 30 minutes. After cell fixation, F-actin was stained with phallodin-Alexa Fluor546 conjugate (red). (**B**) After apical addition of PIP3/Histone H1 complex, cells were fixed and stained for β1-integrin (green), actin (red). (**C**) After apical addition of PIP3/Histone H1 complex, cells were stained for Syndecan-1(green), ZO-1(red). Scale bar is 5 μm.

The PI3-Kinase (PI3K) pathway regulates many cellular processes, including cell metabolism, cell survival, and apoptosis^18^. Phosphatidylinositol-3,4,5-trisphosphate (PIP3), the product of PI3K activity and a key signaling molecule, acts by recruiting proteins that contain PIP3-interacting pleckstrin-homology (PH) domains to cell membranes. In polarized epithelial cells, PIP3 is localized at the basolateral plasma membrane and excluded from the apical plasma membrane, while PIP2 is enriched at the apical membrane. First, the polarized distribution of PIP3 was verified in polarized MDCK cells that were stably transfected with the PIP3 sensor GFP-PH-Akt, i.e., a GFP fusion protein of the PIP3-binding pleckstrin-homology domain of Akt^7^. GFP-PH-Akt localized at the basolateral plasma membrane and partially in the cytoplasm, and was typically absent from the apical membrane (Fig. 1A; left panel). Exogenous addition of PIP3 to the apical plasma membrane domain, through the use of the shuttle protein Histone H1, resulted in the appearance of GFP-PH-Akt in clusters at the apical plasma membrane (Fig. 1A; right panel), indicative for the successful insertion of PIP3 into the apical plasma membrane. The clustered appearance likely reflects their presence in protrusions, as was previously shown by Gassama-Diagne *et al.*^7^ Besides, they showed that basolateral proteins are present within these protrusions, whereas apical proteins are excluded^7^.

In previous work we showed that β1-integrin receptors, that normally mediate cell-cell and cell-ECM contact, play a role in the internalization of non-viral gene delivery vectors by MDCK cells^11^. Likewise, integrins are exploited by viruses to attach to and infect epithelial cells^19^,^20^. Izmailyan and colleagues found that vaccinia virus invasion through β1-integrin activates PI3K/Akt signaling^21^. We investigated whether upon the delivery of exogenous PIP3 to the apical plasma membrane, β1-integrin receptors, that typically localize to the basolateral surface in MDCK monolayers, become exposed at the apical membrane. Figure 1B shows that in control cells β1-integrin (in green) is localized at the basolateral membrane (Fig. 1B; left panel). However, after addition of PIP3/Histone H1 complexes, clusters of β1-integrin were found at the apical surface, as indicated by their localization above the apical plane indicated by actin (Fig. 1B; right panel).

Next, the effect of exogenous PIP3 addition on syndecan-1 localization was investigated. Syndecan-1 is a transmembrane heparan sulfate proteoglycan (HSPG) involved in cell-cell and cell-ECM adhesion, growth factor activation, tumor growth, and microbial infection^22^. Like β1-integrin, syndecans were shown to play a role in the binding of gene vectors^23^,^24^. Specifically, in HeLa cells gene vectors are captured by actin-rich filopodial extensions, while local clustering of filopodia-localized syndecans appear instrumental in their processing to the cell body, which is followed by cellular entry^24^. In control MDCK monolayers, syndecan-1 was mostly present at the basolateral membrane, i.e., below the tight junction (ZO-1) level, and in small apical and cytosolic domains (Fig. 1C; left panel). However, after treatment of MDCK cells with PIP3/histone large clusters of syndecan-1 appeared at the apical membrane (Fig. 1C; right panel). These results demonstrate that the insertion of exogenous PIP3 into the apical surface of polarized MDCK cells induces the redistribution of receptors - previously implicated in host-lipoplex/polyplex interactions - from the basolateral to the apical surface.

### Formation and characterization of PEI/PIP3 polyplexes

In addition to histones, other vectors such as cationic polymers (dendrimers and polyethylenimine (PEI)) have been used for PIP3 delivery to cells^15^. Interestingly, these vectors are also used for nucleic acid delivery, because of their ability to condense nucleic acids (DNA^12^, RNA^25^, oligonucleotides^14^) into nanoscale complexes by electrostatic interaction. Here, we investigated whether PEI/PIP3 polyplexes can be used to enhance the delivery of genetic cargo into polarized epithelial cells through the recruitment of basolateral receptors to the apical membrane, thereby facilitating the subsequent uptake of the gene vector. Fluorescently labeled PEI/PIP3 complexes were spontaneously formed through electrostatic interactions of the negatively charged phosphate groups in DNA and PIP3, with the positively charged amino nitrogen groups in PEI. The particle size and zeta potential of PEI/DNA/PIP3 complexes was 253.2 ± 39.9 nm and 18.6 ± 0.5 mV, respectively (Table 1).

Upon apical incubation of MDCK monolayers with the ternary PEI/DNA/PIP3 complexes, the PEI/DNA/PIP3 particles formed clusters of more than 20 μm in diameter (Supplementary Figure S1B). These large aggregates were not found when PEI/DNA/PIP3 complexes were incubated in cell culture medium in the absence of cells (Supplementary Figure S1A). This suggests that the formation of large aggregates is dependent on the interaction of the particles with the cells. A similar phenomenon has been described for *P. aeruginosa*, which formed large aggregates following interaction with the apical surface of epithelial cells^5^.

### PEI/PIP3 polyplexes deliver PIP3 and recruit basolateral receptors to the apical plasma membrane of MDCK cells

**Figure 2:**
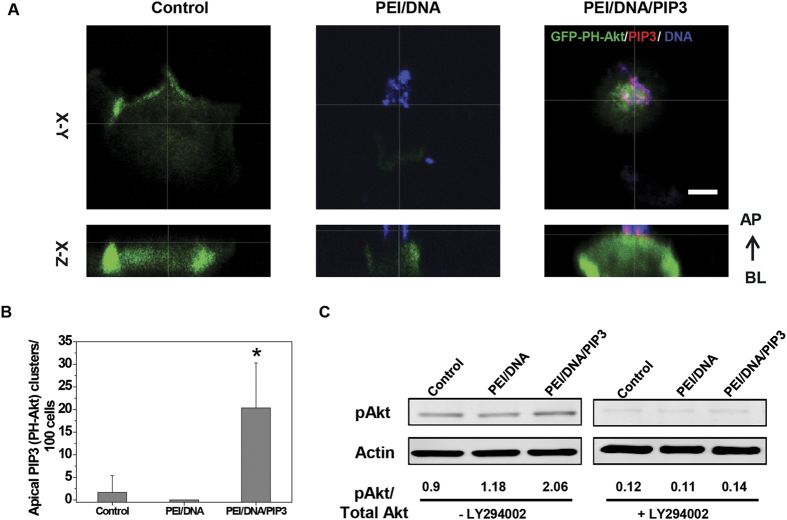
Apical incubation of MDCK cells with PEI/DNA/PIP3 polyplexes leads to PI3-Kinase activation. (**A**) Polarized MDCK cells stably expressing GFP-PH-Akt (green) were treated with PEI/DNA/PIP3 or PEI/DNA complex, or without any treatment (control). Plasmid DNA was labeled by Cy5 (blue), PIP3 was labeled by BODIPY-TMR (red). Scale bar is 5 μm. (**B**) The presence of apical PIP3 (PH-Akt) clusters was quantified from three independent experiments; per condition 80-100 cells were analyzed. Data are presented as mean ± SD. Two-tailed t-test was used to determine statistical difference between each treatment group and control. *p = 0.006. (**C**) MDCK cells were treated with PEI/DNA, and PEI/DNAPIP3 complexes. Cell lysates were analyzed for phosphorylated Akt and total Akt expression by Western blotting. Actin served as a loading control. The numbers below the lanes indicate the phospho-Akt/total Akt ratio.

To determine whether, similar to PIP3/histone (Fig. 1), PEI/PIP3 polyplexes can deliver PIP3 to the apical plasma membrane of polarized epithelial cells, PEI/PIP3 polyplexes were applied to the apical surface of MDCK cell monolayers that stably expressed the PIP3 sensor GFP-PH-Akt. The PEI/PIP3 polyplexes were double-labeled with fluorescent PIP3-Bodipy-TMR and Cy5-pDNA, to visualize their localization. After one hour of incubation, a clear local apical accumulation of GFP-PH-Akt was detected adjacent to the PIP3-Bodipy-TMR (red) signal that colocalized with the Cy5-pDNA (blue) signal, indicating the recruitment of GFP-PH-Akt at the site of PEI/PIP3 polyplex binding to the apical cell surface (Fig. 2A, right panel). In untreated cells, GFP-PH-Akt localized exclusively at the basolateral surface (Fig. 2A, left panel). Also the addition of PEI polyplexes, i.e., without PIP3, to the apical surface did not result in the apical accumulation of GFP-PH-Akt (Fig. 2A, middle panel). In MDCK cell monolayers that were treated with PEI/PIP3 polyplex, on average 20% of the cells showed apical PH-Akt (PIP3) clusters and typically one cluster was observed per cell (Fig. 2B). Furthermore, the addition of PEI/DNA/PIP3 complexes, but not PEI/DNA complexes, to the apical side stimulated the phosphorylation of Akt, which occurred in a PI3-Kinase-dependent manner (Fig. 2C). The latter was shown by the fact that in the presence of the PI3-Kinase inhibitor LY294002, Akt-phosphorylation was effectively inhibited (Fig. 2C). Moreover, LY treatment of MDCK cell monolayers resulted in the redistribution of GFP-PH-Akt from the basolateral membrane to the cytosol (Supplementary Figure S2A). Interestingly, subsequent incubation with PEI/DNA/PIP3 complexes still led to apical accumulation of GFP-PH-Akt at the site of polyplex binding (Supplementary Figure S2B). These data suggest that the transfer of PIP3 from the polyplex to the apical membrane leads to GFP-PH-Akt recruitment and that potentially additional PIP3, arising from the conversion of apical PIP2 into PIP3 due to PI3-Kinase activation, does not play a role in the observed effects. Together, these data indicate that PEI/PIP3 polyplexes successfully deliver PIP3 to the inner leaflet of the apical plasma membrane of polarized MDCK cells.

**Figure 3:**
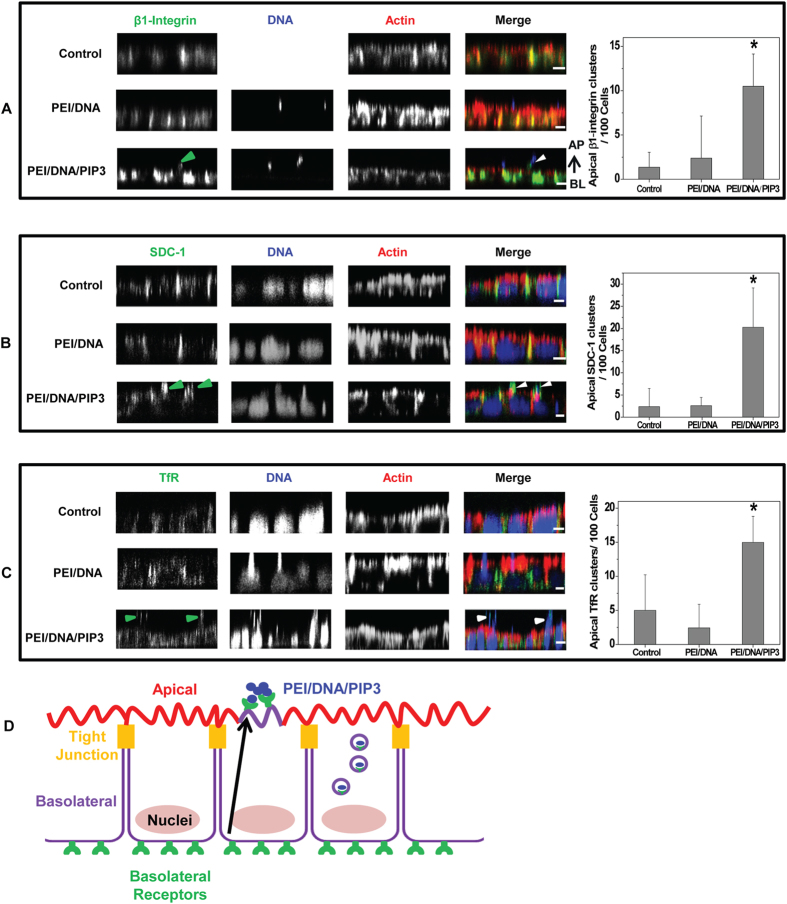
Apical incubation of MDCK cells with PEI/PIP3 polyplexes induces recruitment of basolateral receptors. After apical addition of PEI/DNA/PIP3 or PEI/DNA complex, cells were fixed and immunostained for (**A**) β1-integrin at 1 hr (green). Plasmid DNA in polyplex was labelled by Cy5 (Blue); (**B**) syndecan-1 at 1 hr (green); (**C**) transferrin receptor (TrfR) at 30 min. (green). F-actin was stained with phalloidin-Alexa Fluor 546 (red). Cell nuclei and plasmid DNA were stained with Draq 5 (blue) in (**B,C**): Large round structures underneath apical plane, as indicated by staining for actin, represent nuclei. Irregular clusters above apical plane represent the complexes. Apical appearance of basolateral receptors, that colocalizes with polyplex, is indicated with arrowheads. Scale bar is 5 μm. The presence of apical clusters of β1-integrin, syndecan-1, and TrfR was quantified from at least two independent experiments; per condition 80–100 cells were analyzed. Data are presented as mean ± SD. Two-tailed t-test was used to determine statistical difference between each treatment group and control. *p = 0.0003 (**A**) p = 0.013 (**B**) p = 0.02 (**C**). The cartoon (**D**) illustrates basolateral receptor recruitment by PEI/DNA/PIP3 in MDCK cells.

The recruitment of basolateral receptors upon PIP3 delivery to the apical surface by PEI/PIP3 polyplexes was investigated next. In untreated cells β1-integrin is present at the basolateral plasma membrane (Fig. 3A, upper row). Upon the addition of PEI polyplexes to the apical surface, a limited number of complexes bind to the apical surface, while β1-integrin remains at the basolateral domain (Fig. 3A, middle row). In contrast, the addition of PEI/PIP3 polyplexes (DNA labeled with Cy5, blue) to the apical surface results in more extensive binding of complexes at the apical surface, which at least partially colocalize with β1-integrin (Fig. 3A, bottom row). Similar observations were made for the syndecan-1 and transferrin receptor (TrfR). Specifically, syndecan-1 and TrfR in untreated cells predominantly resided at the basolateral plasma membrane (upper rows in Fig. 3B,C, respectively). The latter is consistent with the 300:1 ratio of basolateral to apical transferrin receptors that was measured in polarized MDCK cells^26^. Addition of PEI polyplexes did not change the distribution of syndecan-1 and transferrin receptors (middle rows in Fig. 3B,C, respectively). However, following treatment of MDCK cell monolayers with PEI/PIP3 polyplexes for one hour, the polyplexes were seen to partially colocalize with apical clusters of syndecan-1 (Fig. 3B, bottom row) and TrfR (Fig. 3C, bottom row). The apical localization of receptor clusters in MDCK cell monolayers can be appreciated from their localization above the apical plane that is indicated by phalloidin-stained actin in the X-Z projection of the MDCK cell monolayer. MDCK cell monolayers that were treated with PEI/PIP3 polyplexes showed on average 10-20 apical receptor clusters per 100 cells, whereas untreated monolayers and monolayers treated with PEI polyplexes showed <5 clusters/100 cells (graphs in Fig. 3A–C). Altogether, the data demonstrate that PEI/PIP3 polyplexes locally deliver PIP3 to the apical plasma membrane which, in turn, mediates the recruitment of basolateral receptors to the site where the polyplexes reside.

### PEI/PIP3 polyplexes show enhanced internalization by MDCK cell monolayers compared to PEI polyplexes

**Figure 4:**
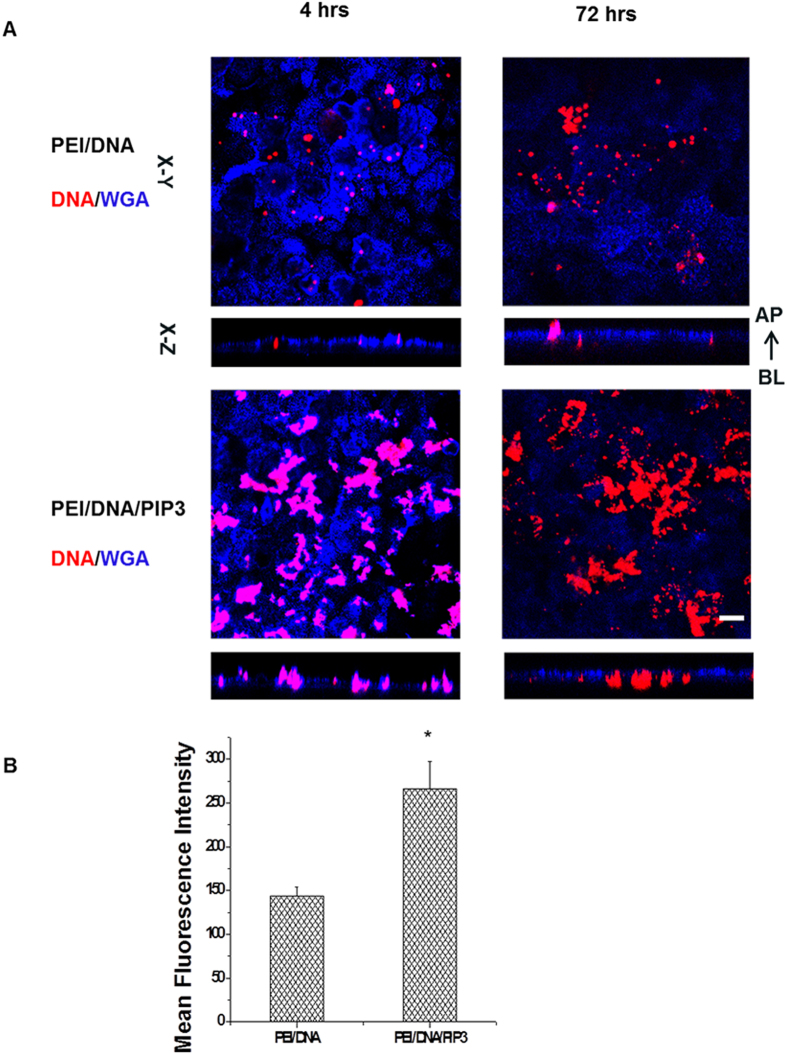
Binding and uptake efficiency of PEI/PIP3 and PEI polyplexes by MDCK cells after 4 hours and 72 hours incubation. (**A**) MDCK cells were incubated with complexes for 4 and 72 h, after which the apical plasma membrane was stained with WGA-Alexa Fluor 633 conjugate (blue). Plasmid DNA was labeled by Cy3 (red). Pink (blue + red) color indicates binding of complexes at the apical plasma membrane. Red color indicates internalization of complexes. Scale bar 10 μm. (**B**) The mean fluorescence intensity of the MDCK cells, representing the fraction of internalized Cy3-labeled polyplexes, was quantified after 72 hours by FACS analysis. Two-tailed t-test was used to determine statistical difference between each treatment group and control. *p = 0.003.

**Figure 5:**
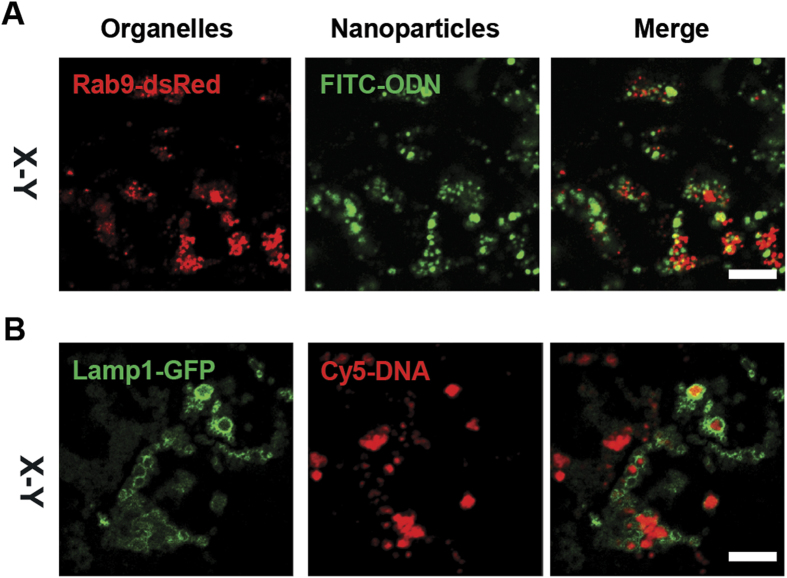
PEI/PIP3 polyplexes localize in late endosomes/lysosomes. (**A**) MDCK cells that transiently express the fluorescent fusion protein Rab9-dsRed (late endosome), and (**B**) Lamp1-GFP (late endosome/lysosome) were treated with PEI/PIP3 polyplexes for 72 hours. Polyplexes were fluorescently labeled with Atto495-ODN (**A**) and Cy5-DNA (**B**) in order to determine colocalization. Scale bar is 5 μm.

The binding and internalization of PEI and PEI/PIP3 polyplexes in MDCK cells was determined using fluorescently labeled polyplexes. Both types of polyplexes were fluorescently labeled through the complexation of Cy-3 DNA, while the apical membrane of MDCK monolayers was stained by a fluorescent wheat germ agglutinin conjugate (WGA-Alexa fluor 633), which selectively binds to N-acetylglucosamine and N-acetylneuraminic acid (sialic acid) residues, in order to discriminate between cell-bound and internalized complexes. After 4 hours of incubation of MDCK cell monolayers with PEI/PIP3 polyplexes an extensive association with the apical surface was detected, as shown by the magenta color, resulting from colocalization of the Cy-3 (red) labeled particles and WGA (blue) (Fig. 4A 4 hrs, lower panel). PEI polyplexes also localized at the apical surface, which is visible from the magenta color, although to a much lesser extent than the PIP3-containing polyplexes (Fig. 4A 4 hrs, compare upper and lower panel). In addition, limited uptake of PEI polyplexes was present at t = 4 h, as indicated by the red fluorescence that localized underneath the apical membrane (Fig. 4A; 4hrs, upper panel). The fact that WGA only stained the apical membrane, and did not penetrate the monolayer to stain the basolateral membrane, indicates that the treatment with PEI and PEI/PIP3 polyplexes did not compromise monolayer integrity, i.e., create imperfections in the monolayer through which WGA could penetrate, and stain the basolateral surface. The non-toxic nature of the treatments was confirmed by MTT assay (Supplementary Figure S3A). After 72 h of incubation of MDCK cells with PEI/PIP3 polyplexes, cell areas were found that showed high internalization of PEI/PIP3 polyplexes (in red) (Fig. 4A; 72 hrs, lower panel). Strikingly, after 72 h of incubation the internalization of PEI polyplexes was comparable to the level detected after 4 h of incubation (Fig. 4A; upper panels), and significantly less than that of the PEI/PIP3 polyplexes. This suggests that the uptake of PEI/DNA reaches its maximum after 4 h of incubation, while the uptake of PEI/DNA/PIP3 occurs more slowly, but is of higher capacity. Quantification of the cellular internalization of PEI and PEI/PIP3 polyplexes at t = 72 h by FACS analysis revealed that the fluorescence intensity per cell was ~2-fold higher in cells incubated with PEI/DNA/PIP3 compared to cells incubated with PEI/DNA (Fig. 4B). At this time point, the PEI/PIP3 polyplexes colocalized with markers of late endosomes (Rab9-dsRed; Fig. 5A), and lysosomes (Lamp1-GFP; Fig. 5B), indicating their uptake via endocytosis. This was confirmed by electron microscopic investigation (Supplementary Figure S4). Electron micrographs of MDCK cell monolayers incubated with PEI/PIP3 polyplexes showed the presence of clusters of polyplexes at the apical membrane, indicated by the presence of microvilli (Supplementary Figure S4A). The morphology of the polyplexes by TEM investigation presents as a toroidal ring (donut-like shape) and shows an internal lamellar-like or fingerprint structure^27^,^28^,^29^. Moreover, the shape of the aggregates at the apical surface as determined by TEM was similar as was shown from the fluorescent images (compare Figure S4A and Fig. 4A). Furthermore, polyplexes were detected within endosomes (Supplementary Figure S4B,C, boxed areas), in lysosomal structures (Supplementary Figure S4D, boxed area), and free in the cytosol (Supplementary Figure S4D, white arrowheads). These data are consistent with the uptake of polyplexes via endocytosis and their processing toward lysosomes.

### PEI/PIP3 polyplexes show efficient endosomal escape

**Figure 6:**
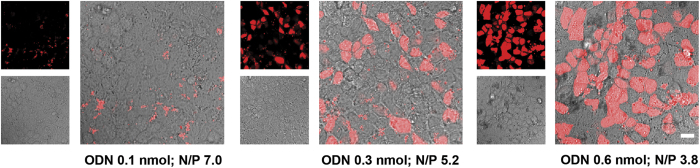
PEI/PIP3 polyplexes mediate efficient endosomal escape of oligonucleotides (ODNs). MDCK cells were incubated for 4 h with PEI/ODN/PIP3 complexes containing 0.1, 0.3, and 0.6 nmol TAMRA-labeled ODNs (red). The overlays of the fluorescent images with the phase contrast images shows the completeness of the MDCK monolayer for each condition. The number of fluorescent nuclei in cells treated with PEI/PIP3 containing 0.6 nmol ODN > PEI/PIP3 containing 0.3 nmol ODN > PEI/PIP3 containing 0.1 nmol ODN ( = 0). Scale bar is 20 μm.

The final step in the transfection process, i.e., the transfer of DNA into the nucleus, is dependent on the temporary absence of the nuclear membrane, that occurs during mitosis^30^. Therefore it is not expected that the uptake of DNA-containing polyplexes in MDCK monolayers will result in gene expression, because polarized cell monolayers show negligible cell division. Indeed, the chromosomal DNA in MDCK monolayers showed essentially no mitotic figures, as was revealed by DAPI staining (data not shown). Notably, because of low cell proliferation in epithelium (e.g. lung) *in vivo* and/or the ‘hidden’ location of the proliferative cells (e.g. in stratum basale in skin epidermis, and in crypts in intestinal epithelium), non-viral delivery systems, including our PIP3-containing polyplexes, are expected to be particularly useful for the delivery of nucleic acids that do not require cell division for their activity, such as antisense oligonucleotides (ODNs) and siRNA. Following their escape from endosomes, ODNs passively diffuse into the nucleus where they can bind to complementary mRNA and inhibit gene expression, while siRNA mediates gene silencing following its binding to the RNA-induced silencing complex (RISC) that is present within the cell’s cytoplasm. Consequently the activity of both ODNs and siRNA is not restricted by the absence of mitotic events. Therefore, while fluorescently labeled DNA was useful to label our nanoparticles in order to investigate cellular binding and uptake of the PEI/PIP3 polyplexes, we used ODNs and siRNA in subsequent experiments to show the potential of our delivery system to induce a physiological effect. Because the endosomal escape of the genetic cargo plays a critical role in determining the eventual transfection efficiency with polyplexes, the endosomal escape of PEI/PIP3 polyplexes was investigated first. In order to visualize the endosomal escape and dissociation of genetic cargo from the polyplexes, fluorescently labeled ODNs were used, because they passively accumulate in the nucleus after their cytosolic release, allowing for easy detection^14^. PEI/PIP3 polyplexes that contained 0.1 nmol ODN (N/P 7) showed significant uptake into MDCK monolayers, but no endosomal escape, as indicated by the punctate fluorescence pattern consistent with the cytoplasmic distribution of endosomes/lysosomes, and the absence of fluorescent nuclei (Fig. 6, left; and Fig. 5A). However, an increase in the amount of ODN in PEI/PIP3 polyplexes (resulting in a concomitant decrease in the N/P ratio), resulted in an efficient nuclear accumulation of ODNs, indicating their efficient endosomal escape (Fig. 6, middle and right). For size and zeta potential of the different complexes, see Table 2. Polarized MDCK monolayers incubated for 4 hrs with PEI/ODN/PIP3 complexes containing 0.3 nmol ODN (N/P ratio 5.3) and 0.6 nmol ODN (N/P ratio 3.8) showed 24.61 ± 4.24% and 56.21 ± 0.91% ODN-positive nuclei, respectively.

### PEI/PIP3 polyplexes induce efficient RNA interference

**Figure 7:**
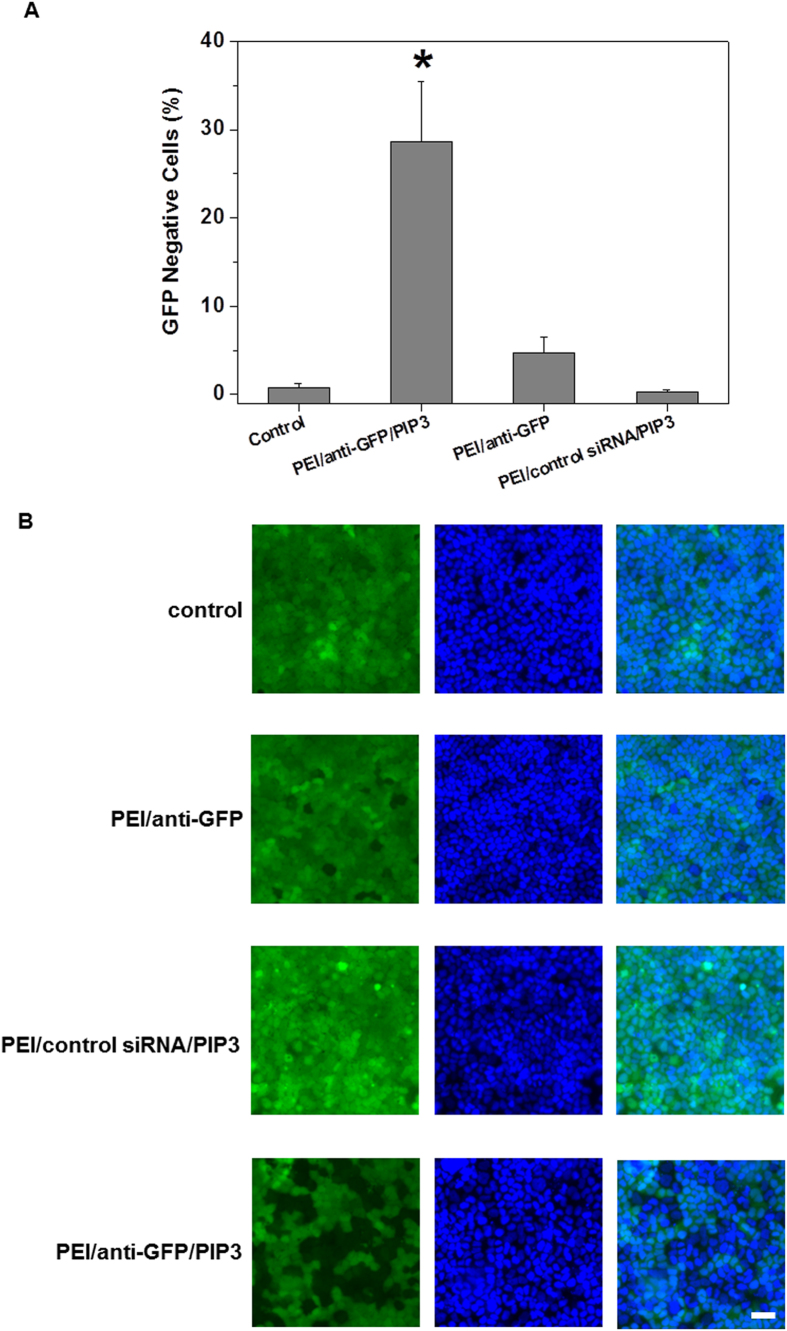
PEI/anti-GFP siRNA/PIP3 complexes mediate efficient gene silencing in MDCK cell monolayers that stably express GFP. Monolayers of GFP-expressing MDCK cells were incubated with PEI/anti-GFP siRNA/PIP3, PEI/anti-GFP siRNA, and PEI/control siRNA/PIP3 complexes for 96 h. (**A**) GFP downregulation was quantified as the number of GFP-negative cells in the MDCK monolayers, using fluorescence microscopy. Results are presented as mean ± SD. Two-tailed t-test was used to determine statistical difference between each treatment group and control. *p = 0.00004 (**B**) Representative images of MDCK-GFP (green) monolayers treated with different complexes are shown, Nuclei were stained with DAPI (blue). scale bar is 30 μm.

Finally, to demonstrate the effectiveness of PEI/PIP3 polyplexes to generate a physiological effect, its ability to induce RNA interference was investigated. To this end, polarized monolayers of MDCK cells stably transfected with GFP, were treated with PEI/PIP3 polyplexes containing 0.1, 0.2, and 0.3 nmol anti-GFP siRNA. Most efficient GFP knockdown was observed in cells treated with PEI/PIP3 polyplexes containing 0.3 nmol siRNA (N/P 3.5). Notably, this N/P ratio is similar to the N/P ratio of PEI/ODN/PIP3 complexes that showed most efficient endosomal escape. Furthermore, the polyplexes exhibited minimal cellular toxicity (Supplementary Figure S3B). For comparison, MDCK monolayers were treated with PEI/anti-GFP siRNA, and PEI/control siRNA/PIP3. In untreated GFP-MDCK monolayers (control) all cells expressed GFP, whereas in monolayers treated with PEI/anti-GFP siRNA/PIP3 ~30% of the cells showed essentially complete knockdown of GFP expression (Fig. 7A). Notably, the fluorescence micrograph of the MDCK monolayer that was treated with PEI/PIP3 polyplexes showed a ‘patchy’ pattern of GFP knockdown (Fig. 7B), that resembles the pattern of binding/uptake of PEI/PIP3 polyplexes (cf. Fig. 4A) and the pattern of their endosomal escape (cf. Fig. 6, right). PEI/PIP3 polyplexes with control siRNA did not lead to a decrease in GFP expression (Fig. 7A,B), which indicates that the observed knockdown is induced by an siRNA-specific effect. Moreover, transfection with PEI/anti-GFP siRNA resulted in less than 5% of GFP-negative cells (Fig. 7A,B), showing the superiority of the PIP3-containing polyplexes in inducing gene silencing.

## Conclusions

Our results indicate that PEI/PIP3 polyplexes are able to insert PIP3 into the apical plasma membrane of polarized MDCK cells; induce apical-basal polarity reversal in these cells; and, promote their cellular internalization, in comparison to PEI polyplexes. Moreover, PEI/PIP3 polyplexes demonstrated efficient endosomal escape and effectiveness in inducing RNA interference.

In conclusion, the PEI/PIP3 polyplex-triggered local apical-basal polarity reversal in epithelial cells, inspired by the pathogenic bacterium *Pseudomonas aeruginosa*, provides a promising opportunity for the entry of drug delivery systems into epithelium without the need for barrier disruption. The transient apical appearance of basolateral receptors solely at the site of polyplex binding likely assures receptor occupancy predominantly by the polyplex, contributing to the safety of the system.

## Methods

### Antibodies, Plasmids and Reagents

Primary antibodies were obtained from the following sources: mouse ZO-1 antibody and rabbit anti-GFP antibody were purchased from Life technologies; mouse β-actin was obtained from Sigma; rabbit Syndecan-1 antibody and mouse Transferrin Receptor antibody were obtained from Invitrogen; rat anti-β1 integrin antibody (AIIB2) was obtained from the Developmental Studies Hybridoma Bank. Anti-mouse, anti-rabbit, and anti-rat Alexafluor^®^555 and Alexafluor^®^488 secondary antibodies were obtained from Life Technologies. Actin filaments were stained with phalloidin–Alexa Fluor 546 (Sigma). Nuclear staining reagent Draq5^®^ was from Cell Signaling Technology and DAPI (4’,6-diamidino-2-phenylindole) from Life Technologies. Alexa Fluor^®^ 633-Wheat Germ Agglutinin was purchased from Life Technologies.

Plasmid DNAs were obtained from the following sources: pEGFP-N1 was purchased from Clontech (USA); pRab9-dsRed, and pLAMP1-GFP were obtained from Addgene (Cambridge, MA, USA). Plasmid DNA encoding the pleckstrin homology (PH) domain of Akt was a gift from dr. Mostov (UCSF/USA). Plasmid DNAs were isolated from transformed E.coli using GenElute TM HP Plasmid Midiprep kits (Sigma Aldrich) following the manufacturer’s protocol. pDNAs were fluorescently labeled with Cy5 or Cy3 using Label IT^®^ Tracker Intracellular Nucleic Acid Localization Kit (Mirus, MA, USA). Branched Polyethyleneimine (PEI; M.W. 25 kDa) was purchased from Sigma Aldrich. Long chain (Di-C16) synthetic phosphoinositides: PtdIns(3,4,5)P3, BODIPY^®^-TMR conjugated PtdIns(3,4,5)P3, and Histone H1 were from Echelon (Salt Lake City, UT). Atto 495-labeled and TAMRA-labeled fully phosphorothioated oligonucleotides (5’-ACTACTACACTAGACTAC-3’) were from Biomers.net GmbH (Ulm, Germany). Anti-GFP siRNA (Sense CAAGCUGACCCUGAAGUUCdTdT and antisense GAACUUCAGGGUCAGCUUGdTdT) was obtained from Biolegio, and negative control siRNA was obtained from Invitrogen.

### MDCK cell culture

MDCK cells were grown in Dulbecco’s modified Eagle’s medium (Gibco, Breda, The Netherlands) containing 10% fetal bovine serum, 2 mM L-glutamine (Gibco), 100 U/ml penicillin (Invitrogen), and 100 mg/ml streptomycin (Invitrogen), at 37 °C and 5% CO2. GFP-PH-Akt MDCK cells were generated by stable transfection of MDCK cells with a plasmid encoding the pleckstrin homology (PH) domain of Akt. For experiments MDCK cells were plated at 1 × 10^5^ cells/well in 12-well Transwell filter plates from Costar (Corning Life Sciences, Acton, MA). The next day, the cell culture medium was refreshed. At day 3 after plating, cell resistance was measured with a Millicell-ERS device (Millipore, Billerica, MA) and experiments were performed only if TEER > 178 Ω/cm^2^.

### Phosphoinositides delivery to MDCK cells by Histone H1

Complexes of phosphoinositides (PtdIns(3,4,5)P3) and Histone H1 were made according to the manufacturer’s protocol. Briefly, 10 μL of a 300 μM phosphoinositide solution (PBS, pH 7.4) was added to 10 μL of 100 μM histone H1 (water), gently mixed by pipetting, and incubated for 10 minutes at room temperature. The resulting complexes were diluted into 100 μL medium and added to the apical side of the monolayer of MDCK cells and incubated for different time periods.

### Preparation of PEI/DNA and PEI/DNA/PIP3 polyplexes

**Figure 8:**
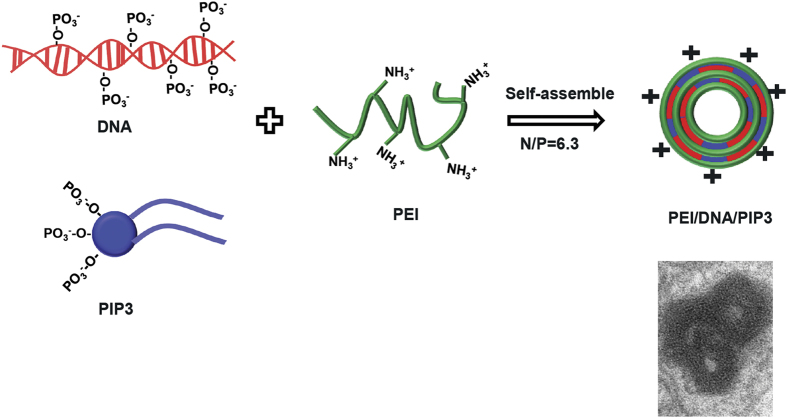
Formation of PEI/DNA/PIP3 ternary complexes. N/P = (nitrogen groups in PEI)/(phosphate groups in DNA and PIP3).

Branched PEI 25 kDa is considered as one of the most potent synthetic gene carriers *in vitro*. Here it was used as the polycation in the formation of a ternary polyplex formulation. Phosphoinositide-containing PEI polyplexes were prepared as shown in Fig. 8. Briefly, 10 μL 300 μM phosphoinositides in PBS (pH 7.4) was mixed with 1 μg of (pEGFP-N1) DNA in 0.1 mL serum-free medium by gentle pipetting. Then branched PEI (200 μg/mL) was added to the DNA/PIP3 mixture and rapidly mixed by pipetting, to obtain PEI/DNA/PIP3 complexes at an N/P ratio of 6.3, where N represents polymer amino groups and P comprises phosphate groups originating from the DNA and the phospholipids. The resulting mixture was incubated for 20 min at ambient temperature to yield the PEI/DNA/PIP3 ternary polyplex. PEI/DNA complexes were made by directly mixing PEI stock solution and DNA in serum-free medium at N/P ratio of 10, where N represents polymer amino groups and P represents DNA phosphate groups. The particle size and zeta potential were measured using a Malvern Zetasizer NS90 (Malvern Instruments, Malvern,UK). The N/P ratio of 10 was used for PEI/DNA complexes because at this ratio optimal transfection of subconfluent MDCK cells was obtained with minimal toxicity. For PEI/DNA/PIP3 complexes the same amount of PIP3 was used as was shown to be effective in recruiting basolateral receptors when complexed with histon H1. The amount of DNA was kept constant between the two types of particles. PEI was added to completely complex all DNA, and yield similar transfection efficiency in subconfluent MDCK cells as PEI/DNA complexes.

### Western blot analysis

Polarized MDCK cell monolayers were treated with PEI/DNA and PEI/DNA/PIP3 polyplexes for 4 h with or without prior treatment with the PI3 kinase inhibitor LY294002 (20 μM, 30 min). Cells on the filter were lysed in 150 μL ice-cold 2 × SDS-Laemlli buffer, heated for 5 min at 95 °C, and subjected to SDS-PAGE and Western blotting following standard procedures. Primary antibodies used were rabbit anti-phospho-Akt Ser473 (Cell Signaling, 1:1000), rabbit anti-Akt (Cell Signaling, 1:1000) and mouse anti-β-Actin (Sigma-Aldrich, 1:2000). Alexafluor^®^ secondary antibodies were used. The signals were detected using the Odyssey Infrared Imaging System (Li-Cor Biosciences, Lincoln, NE) and analyzed with Image-J software. The experiment was repeated three times.

### Transfection of MDCK cell monolayers with polyplexes

MDCK cell monolayers were rinsed twice with warm phosphate buffered saline (HBSS, pH 7.4). Subsequently, 0.4 ml of serum-free medium and 0.1 ml of PEI/DNA/PIP3 ternary complexes or PEI/DNA complexes were added to the apical surface of the MDCK cells. The final DNA concentration was 1.0 μg/well. At different time-points the cells were fixed with 4% paraformaldehyde in PBS and processed for immunostaining, as described below. Alternatively, for quantification of the cellular uptake, the cells were supplied with 0.4 ml complete culture medium after transfection for 4 h. After 72 h of incubation the cells were rinsed twice with PBS. Subsequently cells were treated with trypsin/EDTA for 8 min, collected by centrifugation, suspended in 0.4 ml PBS and kept on ice until analysis. The percentage of Cy5-positive cells was analyzed by flow cytometry using a FACS-Calibur Instrument (Becton-Dickinson).

### Immunofluorescence staining and image analysis

After fixation, cells were rinsed with 10 mM glycine in 0.1% BSA in PBS, permeabilized with 0.1% Triton X-100 in PBS, incubated with primary antibody at 37 °C for 1 h, and incubated with fluorophore-conjugated secondary antibody. Filamentous actin was visualized by incubating samples with fluorophore-conjugated phalloidin. Cell nuclei were stained by the DNA probes Draq5^®^ and DAPI. Alexa Fluor^®^ 633-conjugated Wheat Germ Agglutinin (to stain the apical plasma membrane) was used according to the manufacturer’s protocol. The samples were investigated by confocal microscopy using a Leica SP2 AOBS Confocal microscope or a Leica SP8 Confocal microscope. Images were analyzed with Imaris software (Bitplane).

### Cell viability assay

To evaluate whether PEI/DNA, PEI/DNA/PIP3, and PEI/siRNA/PIP3 induced cytotoxicity in MDCK cells, an MTT colorimetric assay was performed. Briefly, MDCK cells were seeded in 96-well plates at a density of 5000 cells/well. After 72 h, when the cells had formed a monolayer, the cells were incubated with the polyplexes in serum-free medium for 24 h, after which the complexes were aspirated and complete medium was added. After another 48 h, 20 μl MTT in 5 mg/mL phosphate buffered saline solution was added to each well. After 4 h of incubation at room temperature, the supernatant was aspirated and the formazan crystals were dissolved in 180 μL DMSO. For siRNA-containing polyplexes, the cells were incubated for 96 h with the different siRNA complexes, and the medium was refreshed every 24 h. Absorption was measured photometrically at 570 nm with a background (serum-free medium plus MTT) correction using a Bio-Tek μQuant™ Microplate Spectrophotometer. Values of 4 measurements were normalized to 100% for the control group (cells exposed to serum-free medium without complexes). The cell viability was calculated by the formula: (Absorbance /Absorbance (control)) × 100%.

### Transmission electron microscopy of transfected MDCK cells

Polarized MDCK cells grown on transwells were incubated with PEI/DNA/PIP3 and PEI/DNA complexes for 4 h and 72 h. Cells were fixed for 1 hour on ice in 1.5% glutaraldehyde in 0.1 M cacodylate buffer, pH 7.4, containing 1% sucrose. After postfixation in 1% OsO_4_/1.5% K_4_Fe(CN)_6_, cells were dehydrated in graded alcohol series and embedded in Epon 812. After polymerization for 4 days at 45 °C, ultra-thin sections were cut and stained with 1% tannic acid and 1% uranylacetate. (All chemicals used for the processing of cells for investigation by transmission electron microscopy were from Sigma). The sections were examined using a Philips CM 100 electron microscope (Eindhoven, The Netherlands) operating at 60 kV, and micrographs were taken.

### Endosomal escape of PEI and PEI/PIP3 polyplexes

Polyplexes containing TAMRA-ODN were used to allow for direct quantification of the endosomal escape of the polyplexes, by evaluating the nuclear accumulation of ODNs. MDCK monolayers were grown on Lab-TekIIchamber slides (Thermo Scientific) after which PEI/TAMRA-ODN/PIP3 or PEI/TAMRA-ODN polyplexes, containing 0.1, 0.3, and 0.6 nmol ODN, were added to the apical side of the MDCK cell monolayer. After 4 h of incubation, the monolayers were rinsed with HBSS, and of each condition three randomly selected areas were imaged by confocal microscopy. The percentage of release was calculated as: TAMRA-ODN positive nuclei/ total cell nuclei.

### RNA interference with PEI/PIP3 polyplexes

PEI/anti-GFP siRNA/PIP3 complexes were prepared following the same protocol as for PEI/DNA/PIP3 complexes. MDCK cells stably expressing GFP were grown as a polarized monolayer. Cells were incubated with PEI/siRNA and PEI/siRNA/PIP3 complexes containing 0.3 nmol anti-GFP siRNA or negative control siRNA for 96 hrs. GFP down-regulation in the cell monolayers was quantified as the percentage of GFP-negative cells. GFP protein was detected by rabbit anti-GFP (Life Technologies, 1:1000), mouse anti-β-Actin (Sigma-Aldrich, 1:2000) was used as loading control. The experiment was repeated twice.

### Statistical analysis

Data are expressed as mean ± standard deviation (SD) and were obtained from at least two independent experiments. Statistical analysis was performed using the two-tailed t-test. *p* < 0.05 was considered significant.

## Additional Information

**How to cite this article**: Wang, C. *et al.* Entry of PIP3-containing polyplexes into MDCK epithelial cells by local apical-basal polarity reversal. *Sci. Rep.* **6**, 21436; doi: 10.1038/srep21436 (2016).

## Supplementary Material

Supplementary Information

## Figures and Tables

**Table 1:** The particle size and zeta potential of PEI/DNA (N/P 10) and PEI/DNA/PIP3 (N/P 6.3).

	Particle size (nm)	Zeta potential (mV)
PEI/DNA/PIP3	253.2 ± 39.9	18.6 ± 0.5
PEI/DNA	92.4 ± 1.0	18.3 ± 1.9

**Table 2:** Particle size and zeta potential of PEI/PIP3 complexes with ODN.

	Particle Size (nm)	Zeta potential (mV)
PEI/ODN/PIP3 (ODN 0.1 nmol; N/P 7.0)	133.5 ± 0.8	40.3 ± 0.4
PEI/ODN/PIP3 (ODN 0.3 nmol; N/P 5.2)	120.0 ± 0.7	37.5 ± 0.7
PEI/ODN/PIP3 (ODN 0.6 nmol; N/P 3.8)	127.8 ± 0.8	18.7 ± 0.8

Different amounts of ODN (0.1 nmol, 0.3 nmol, 0.6 nmol) were complexed by PEI/PIP3, resulting in polyplexes with N/P ratios of 7.0, 5.2, and 3.8, respectively.
