# Supplementary material for: Entry of PIP3-containing polyplexes into MDCK epithelial cells by local apical-basal polarity reversal
Source: Sci Rep. 2016 Feb 22;6:21436. doi: 10.1038/srep21436 (PMC4761886; doi:10.1038/srep21436)
Supplement: Supplementary Information [file srep21436-s1.doc]

Supplementary information

**Entry of PIP3-containing polyplexes into MDCK epithelial cells by local apical-basal polarity reversal**

Cuifeng Wang, Edwin de Jong, Klaas A. Sjollema, Inge S. Zuhorn^＊^

**
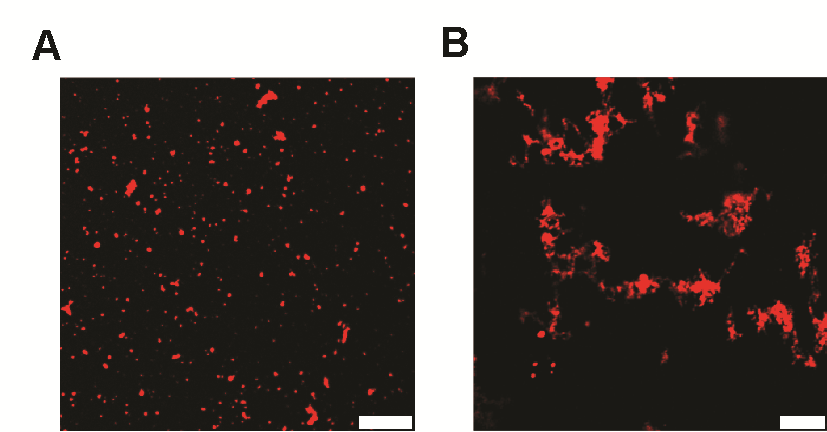
**

**Supplementary Figure S1.** Relative particle sizes of PEI/DNA/PIP3 polyplexes in the absence and presence of MDCK cells. A, The morphology of PIP3-containing polyplexes following incubation in the absence of MDCK cells for 4 h at 37°C. B, The morphology of PIP3-containing particles following incubation in the presence of MDCK cells for 4 h at 37°C. Particles are fluorescently labeled with Cy3- DNA (red); scale bar is 10 µm.

**
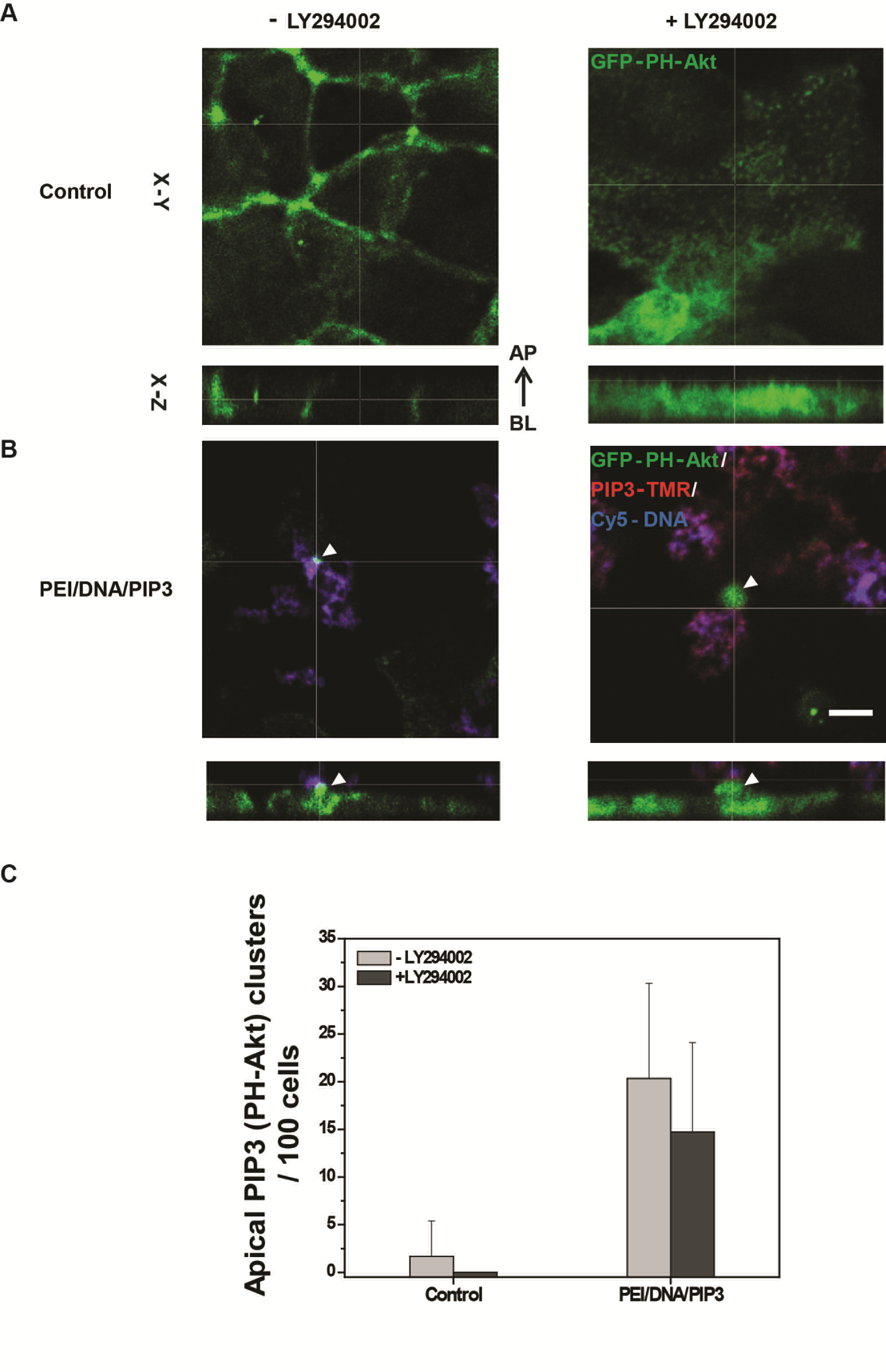
**

**Supplementary Figure S2. Recruitment of GFP-PH-Akt to the apical side of MDCK cells by PEI/DNA/PIP3 complexes is independent of PI3-Kinase activity.** In control MDCK cells GFP-PH-Akt localizes at the basolateral membrane (Figure S2A, left panel). In the presence of LY294002 GFP-PH-Akt localizes in the cytosol (Figure S2A, right panel). Both in the absence and presence of LY294002, PEI/DNA/PIP3 complexes (double-labeled with PIP3-TMR (red) and Cy5-DNA (blue)) induce recruitment of GFP-PH-Akt to the apical side of MDCK cells (Figure S2B, white arrowheads). Scale bar is 5 µm. B, The presence of apical PIP3 (PH-Akt) clusters was quantified from two independent experiments. Data are presented as mean ± SD.

**
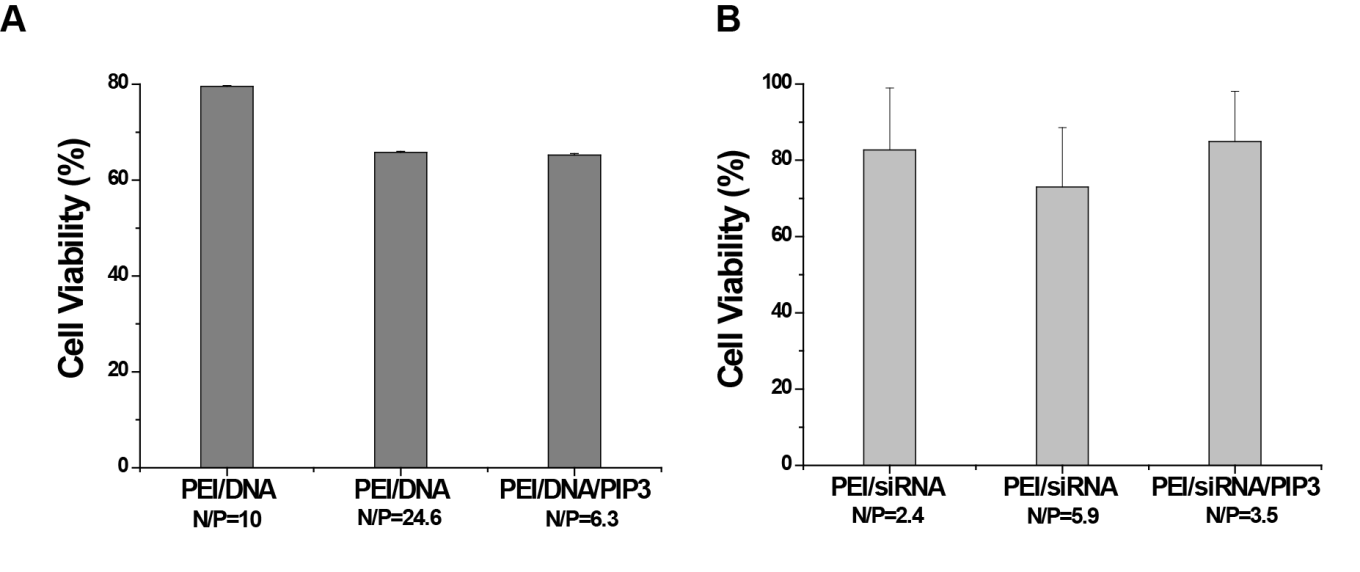
**

**Supplementary Figure S3. Cell viability of MDCK cells after incubation with PEI and PEI/PIP3 complexes, as determined by MTT cytotoxicity test.** A, MDCK cells grown in a 96-well plates were treated with PEI/DNA (N/P=10) and PEI/DNA/PIP3 (N/P=6.3) polyplexes. Because PEI/DNA/PIP3 contains a higher amount of PEI than PEI/DNA, the contribution of this higher amount of PEI to cellular cytotoxicity was investigated as well. To this end, cells were treated with PEI/DNA complexes containing the same amount of PEI as used in PEI/DNA/PIP3, i.e., PEI/DNA complexes with an N/P of 24.6. The cells were refreshed with medium every 24 hours. At 72 hours after the addition of the complexes, the mitochondrial metabolic activity was assessed by MTT assay. B, MDCK-GFP cell monolayers were treated with PEI/anti-GFP siRNA and PEI/anti-GFP siRNA/PIP3 complexes containing 0.3 nmol siRNA. The cells were refreshed with medium every 24 hours. After a total of 96 hours, the MTT assay was performed. Values represent the mean ± SD of three independent experiments performed in triplicate.

**
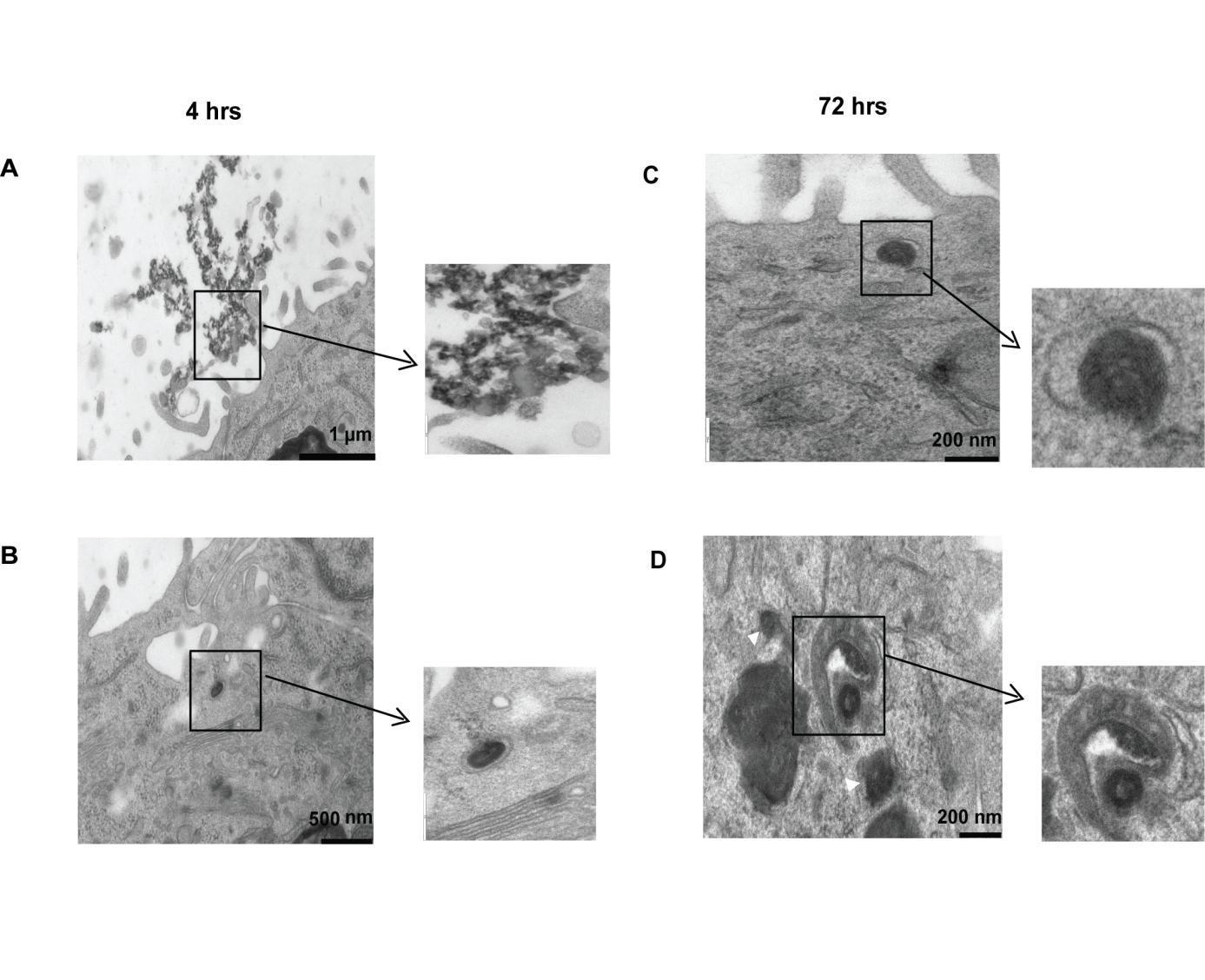
**

**Supplementary Figure S4. Ultrastructural investigation of the intracellular fate of PEI/PIP3 polyplexes in MDCK cells by Transmission Electron Microscopy.** MDCK cells were incubated with PEI/DNA/PIP3 complex for 4 h (A, B) or 72 h (C,D). Electron-dense fingerprint-like structures, representing PEI/DNA/PIP3 complex, were present A, at the apical cell surface, as exemplified by the presence of microvilli; B, C, within endosomal structures; and D, in multilamellar structures (boxed area) and free in the cytosol (white arrowheads).
